# Supplementary material for: Content-rich biological network constructed by mining PubMed abstracts
Source: BMC Bioinformatics. 2004 Oct 8;5:147. doi: 10.1186/1471-2105-5-147 (PMC528731; doi:10.1186/1471-2105-5-147)
Supplement: Additional File 2 — The original results of the above study (non-essential files are deleted to keep the file size under the limit set by BMC bioinformatics). [file 1471-2105-5-147-S2.bz2 › chilibotAdditionalFile2/dip40/311ID8405064E1360/html/TNFSF9_TNFRSF9.html]

 


 **TNFSF9** and **TNFRSF9** 
  
Found 93 abstracts in PubMed, retrieved 40.  
 

 What does Google say? 
 PDF only 
| .edu only 

---

**Interactive relationship** (e.g. stimulation, inhibition, etc)

**Stimulatory relationship**- Soluble 4 1BBL  [ **TNFSF9** ]  was active in that it competed with recombinant 4 1BBL  [ **TNFSF9** ]  for binding to the 4 1BB  [ **TNFRSF9** ]  receptor and was able to costimulate IL 2 and IFN gamma release from peripheral T cells.  Ref: 11564827 J Immunol, 2001
- 4 1BB  [ **TNFRSF9** ]  ligand  [ **TNFSF9** ]  4 1BBL  [ **TNFSF9** ] , a member of the tumor necrosis factor  [ **TNFSF9** ]  TNF superfamily, interacts with 4 1BB  [ **TNFRSF9** ]  CDw137 expressed on activated T cells and delivers a costimulatory signal for T cell activation and growth.  Ref: 12594573 Cancer Immunol Immunother, 2003
- 4 1BB  [ **TNFRSF9** ]  cross linking, either by binding to 4 1BBL  [ **TNFSF9** ]  or by antibody ligation, delivers a costimulatory signal to enhance T cell activation and proliferation.  Ref: 11280763 Cancer Res, 2001
- 4 1BB  [ **TNFRSF9** ]  is a member of the tumor necrosis factor  [ **TNFSF9** ]  receptor superfamily and provides a costimulatory signal by interaction with 4 1BB  [ **TNFRSF9** ]  ligand  [ **TNFSF9** ]  expressed on antigen presenting cells.  Ref: 12706838 FEBS Lett, 2003
- Initially, scientific interest in the 4 1BB  [ **TNFRSF9** ]  4 1BB  [ **TNFRSF9** ]  Ligand  [ **TNFSF9** ]  system focused on the role of the 4 1BB  [ **TNFRSF9** ]  CD137  [ **TNFRSF9** ]  receptor in the costimulation of T cells.  Ref: 12467303 Int J Clin Pharmacol Ther, 2002
- CD4 and CD8 TCR transgenic T cells up regulate 4 1BB  [ **TNFRSF9** ] , OX40, and CD27 and respond to 4 1BBL  [ **TNFSF9** ]  mediated costimulation during a primary response to peptide Ag.  Ref: 11466348 J Immunol, 2001
- An increase in the caspase activities was also observed in the HeLaTR cells transfected with 4 1BB  [ **TNFRSF9** ]  cDNA, and the 4 1BB  [ **TNFRSF9** ]  Ligand  [ **TNFSF9** ]  further increased the caspase activities of the HeLaTR cells overexpressing the 4 1BB  [ **TNFRSF9** ] .  Ref: 12846840 Eur J Biochem< MedlineTA>Eur J Biochem, 2001
- Recent studies highlight the participation of 4 1BB  [ **TNFRSF9** ]  and its ligand 4 1BBL  [ **TNFSF9** ]  in a more complex network of immune cell responses and suggest that intervening in the 4 1BB  [ **TNFRSF9** ]  costimulatory pathway might have some potential as a therapeutic approach to immune disorders.  Ref: 12133793 Trends Immunol, 2002
- 4 1BB  [ **TNFRSF9** ]  CD137  [ **TNFRSF9** ]  is induced on activated CD4 and CD8 T cells and delivers a costimulatory signal upon binding the 4 1BB  [ **TNFRSF9** ]  ligand  [ **TNFSF9** ]  4 1BBL  [ **TNFSF9** ]  expressed on antigen presenting cells.  Ref: 12496166 Infect Immun, 2003
- Cleavage of 4 1BBL  [ **TNFSF9** ]  to an active soluble form would alter both proximal and distal cellular responses, including cell survival and costimulatory or inflammatory responses, that are mediated through the 4 1BB  [ **TNFRSF9** ]  pathway.  Ref: 11564827 J Immunol, 2001
**Neutral relationship**- Blocking 4 1BB  [ **TNFRSF9** ]  4 1BB  [ **TNFRSF9** ]  Ligand  [ **TNFSF9** ]  Interactions Prevents Herpetic Stromal Keratitis.  Ref: 12847221 J Immunol, 2003
- We used the murine model of HSK to demonstrate the importance of the interaction between an inducible T cell costimulatory receptor, 4 1BB  [ **TNFRSF9** ] , and its ligand, 4 1BB  [ **TNFRSF9** ]  ligand  [ **TNFSF9** ]  4 1BBL  [ **TNFSF9** ] , in the development of this disease.  Ref: 12847221 J Immunol, 2003
- The interaction of 4 1BB  [ **TNFRSF9** ]  and 4 1BB  [ **TNFRSF9** ]  ligand  [ **TNFSF9** ]  provides a costimulatory signal leading to T cell activation.  Ref: 12460192 Immunology, 2002
- By quantifying graft vs host disease alloresponses in vivo, we demonstrate that both CD4 and CD8 T cell mediated alloresponses are regulated by 4 1BB  [ **TNFRSF9** ]  4 1BB  [ **TNFRSF9** ]  ligand  [ **TNFSF9** ]  interactions to approximately the same extent.  Ref: 11207270 J Immunol, 2001
- For maintenance and full expansion of CTL, interaction of the DC expressed 4 1BB  [ **TNFRSF9** ]  ligand  [ **TNFSF9** ]  with its receptor 4 1BB  [ **TNFRSF9** ]  on CTL is also important.  Ref: 12445291 Immunol Rev, 2002
- Furthermore, LPS mediated growth and survival of superantigen SAg stimulated T cells appeared to be partially dependent on interactions between 4 1BB  [ **TNFRSF9** ]  and 4 1BB  [ **TNFRSF9** ]  ligand  [ **TNFSF9** ]  4 1BBL  [ **TNFSF9** ] .  Ref: 11306146 Immunol Lett, 2001
- Interactions between 4 1BB  [ **TNFRSF9** ]  and its ligand, 4 1BBL  [ **TNFSF9** ] , enhance CD8 T cell mediated antiviral and antitumor immunity in vivo.  Ref: 12356681 Int Immunol, 2002
- These data are the first to suggest a stimulatory role for endogenous 4 1BB  [ **TNFRSF9** ]  4 1BBL  [ **TNFSF9** ]  interactions during a humoral immune response to a pathogen and further underscore significant differences in costimulation requirements for an in vivo protein versus polysaccharide specific Ig isotype response to an extracellular bacterium.  Ref: 12496166 Infect Immun, 2003
- These results indicate that the release of soluble 4 1BBL  [ **TNFSF9** ]  from the cell surface is mediated by one or more sheddases and likely regulates 4 1BB  [ **TNFRSF9** ]  4 1BBL  [ **TNFSF9** ]  interactions between cells in vivo.  Ref: 11564827 J Immunol, 2001
- Interaction of 4 1BB  [ **TNFRSF9** ]  CD137  [ **TNFRSF9** ]  and its ligand 4 1BBL  [ **TNFSF9** ]  is thought to positively regulate cell mediated and humoral immune responses.  Ref: 11509610 J Immunol, 2001
- In BALB c mice, HSK ordinarily induced by infection with the RE strain of herpes was prevented by blocking 4 1BB  [ **TNFRSF9** ]  4 1BBL  [ **TNFSF9** ]  interaction, either by deleting 4 1BB  [ **TNFRSF9** ]  in mutant 4 1BB  [ **TNFRSF9** ]  mice or by introducing mAbs against 4 1BBL  [ **TNFSF9** ] .  Ref: 12847221 J Immunol, 2003
- To further characterize the expression and function of 4 1BBL  [ **TNFSF9** ] , we newly generated an anti mouse 4 1BBL  [ **TNFSF9** ]  mAb TKS 1, which can inhibit the interaction of 4 1BBL  [ **TNFSF9** ]  with 4 1BB  [ **TNFRSF9** ] .  Ref: 11867564 Int Immunol, 2002
- Strong cytotoxicity of memory type CTLs to a 4 1BB  [ **TNFRSF9** ]  ligand  [ **TNFSF9** ]  expressing Epstein Barr virus EBV transformed B cell line was almost completely abrogated by 4 1BB  [ **TNFRSF9** ]  Fc, a soluble form of 4 1BB  [ **TNFRSF9** ] , suggesting involvement of 4 1BB  [ **TNFRSF9** ]  in cytolytic processes.  Ref: 12384425 Blood, 2002

**Non-interactive relationship** (e.g. studied together, co-existance, homology, etc.)

- Flow cytometric analyses using TKS 1 and an anti mouse 4 1BB  [ **TNFRSF9** ]  mAb indicated that 4 1BB  [ **TNFRSF9** ]  was inducible on both CD4 and CD8 splenic T cells by stimulation with immobilized anti CD3 mAb, but 4 1BBL  [ **TNFSF9** ]  was not expressed on resting or activated T cells.  Ref: 11867564 Int Immunol, 2002
- We investigated a potential role for 4 1BB  [ **TNFRSF9** ] , a costimulatory molecule structurally related to members of the tumor necrosis factor  [ **TNFSF9** ]  TNF receptor family, in the generation and functional activation of CD28 CTLs by using human cord blood CB cells composed exclusively of naive CD8 T cells with few or no CD28 CTLs.  Ref: 12384425 Blood, 2002
- its ligand, 4 1BB  [ **TNFRSF9** ]  ligand  [ **TNFSF9** ]  is expressed on APCs.  Ref: 11207270 J Immunol, 2001
- To investigate the roles of the co stimulatory molecules CD30 CD30L, CD27 CD27L, OX40 OX40L, and 4 1BB  [ **TNFRSF9** ]  4 1BBL  [ **TNFSF9** ] , which belong to the tumor necrosis factor  [ **TNFSF9** ]  TNF receptor ligand superfamily, in the development of acute viral myocarditis, the expression of these molecules was first analysed in the hearts of mice with acute myocarditis induced by Coxsackievirus B3 CVB3 in vivo.  Ref: 11745696 J Pathol, 2001
- Provision of 4 1BB  [ **TNFRSF9** ]  ligand  [ **TNFSF9** ]  enhances effector and memory CTL responses generated by immunization with dendritic cells expressing a human tumor associated antigen.  Ref: 12626542 J Immunol, 2003
- Although some reports have suggested that 4 1BB  [ **TNFRSF9** ]  is primarily involved in CD8 T cell activation, in this report we demonstrate that both CD4 and CD8 T cells respond to 4 1BB  [ **TNFRSF9** ]  ligand  [ **TNFSF9** ]  4 1BBL  [ **TNFSF9** ]  with similar efficacy.  Ref: 11466348 J Immunol, 2001
- Polyclonal CD4 T lymphocytes can be activated and expanded using anti CD3 and anti CD28 antibodies presented on magnetic beads, and CD8 T lymphocytes can be successfully expanded using a novel genetically engineered cell based technology that presents anti CD3 and anti CD28 along with the costimulatory molecule CD137  [ **TNFRSF9** ]  4 1BBL  [ **TNFSF9** ] .  Ref: 12857980 Immunol Res, 2003
- ... 4 1BB  [ **TNFRSF9** ]  ligand  [ **TNFSF9** ]  4 1BBL  [ **TNFSF9** ]  mice show no defect in primary T cell expansion to influenza virus.  Ref: 11937529 J Immunol, 2002
- In this study, we compared the effect of a systemically delivered agonistic anti 4 1BB  [ **TNFRSF9** ]  monoclonal antibody anti 4 1BB  [ **TNFRSF9** ]  mAb with intra tumoral adenoviral mediated gene transfer of the 4 1BB  [ **TNFRSF9** ]  ligand  [ **TNFSF9** ]  ADV 4 1BBL  [ **TNFSF9** ]  to liver metastases in a syngeneic animal model of breast cancer.  Ref: 12040460 Gene Ther, 2002
- Although treatment of the HeLaTR cells with T3 did not induce expression of NF kappaB reporter luciferase, even in the presence of the 4 1BB  [ **TNFRSF9** ]  Ligand  [ **TNFSF9** ] , it increased the caspase activities.  Ref: 12846840 Eur J Biochem< MedlineTA>Eur J Biochem, 2002
- When combined with intra tumoral delivery of the IL 12 gene, both anti 4 1BB  [ **TNFRSF9** ]  mAb and ADV 4 1BBL  [ **TNFSF9** ]  were synergistic and led to survival rates of 87% and 78%, respectively.  Ref: 12040460 Gene Ther, 2002
- The anti tumor immunity is mainly mediated by CD4 T cells in IL 12 plus 4 1BB  [ **TNFRSF9** ]  ligand  [ **TNFSF9** ]  treated animals, and CD8 T cells in IL 12 plus anti 4 1BB  [ **TNFRSF9** ]  mAb treated animals.  Ref: 12040460 Gene Ther, 2002
- Expression and function of 4 1BB  [ **TNFRSF9** ]  and 4 1BB  [ **TNFRSF9** ]  ligand  [ **TNFSF9** ]  on murine dendritic cells.  Ref: 11867564 Int Immunol, 2002
- Soluble CD137  [ **TNFRSF9** ]  4 1BB  [ **TNFRSF9** ]  ligand  [ **TNFSF9** ]  is released following leukocyte activation and is found in sera of patients with hematological malignancies.  Ref: 11564827 J Immunol, 2001
- A novel adenovirus expressing human 4 1BB  [ **TNFRSF9** ]  ligand  [ **TNFSF9** ]  enhances antitumor immunity.  Ref: 12594573 Cancer Immunol Immunother, 2003
- Temporal segregation of 4 1BB  [ **TNFRSF9** ]  versus CD28 mediated costimulation 4 1BB  [ **TNFRSF9** ]  ligand  [ **TNFSF9** ]  influences T cell numbers late in the primary response and regulates the size of the T cell memory response following influenza infection.  Ref: 11937529 J Immunol, 2002
- ... 4 1BB  [ **TNFRSF9** ]  4 1BBL  [ **TNFSF9** ]  sustains both CD4 and CD8 T cell responses, as well as enhances cell division and T cell effector function.  Ref: 11466348 J Immunol, 2001
- 4 1BB  [ **TNFRSF9** ]  Ligand  [ **TNFSF9** ]  Stimulation Enhances Myeloid Dendritic Cell Maturation from Human Umbilical Cord Blood CD34 Progenitor Cells.  Ref: 12590704 J Hematother Stem Cell Res, 2002
- 4 1BB  [ **TNFRSF9** ]  CDw137 and its ligand 4 1BBL  [ **TNFSF9** ]  have been implicated in cellular immune responses.  Ref: 11867564 Int Immunol, 2002
- These subsets differed in their expression of DC associated differentiation activation antigens and co stimulator molecules including CD83, CMRF 44, CMRF 5 7, CD86, and 4 1BB  [ **TNFRSF9** ]  ligand  [ **TNFSF9** ] .  Ref: 11438475 Am J Pathol, 2001
- We examined the effect of gene transfer of the 4 1BB  [ **TNFRSF9** ]  ligand  [ **TNFSF9** ]  4 1BBL  [ **TNFSF9** ]  into NRS1 cells.  Ref: 11122458 Immunology, 2000
- CD28 and 4 1BB  [ **TNFRSF9** ]  were found to synergize in the induction of IL 2 by human T cells, and CTLA Ig partially blocked 4 1BBL  [ **TNFSF9** ]  dependent IL 2 production.  Ref: 11994439 J Immunol, 2002
- In this review we discuss the functional properties of 4 1BB  [ **TNFRSF9** ]  Ligand  [ **TNFSF9** ]  such as its preference for CD8 positive T cells and the differences to costimulation via the B7 CD28 system.  Ref: 12467303 Int J Clin Pharmacol Ther, 2002
- 4 1BB  [ **TNFRSF9** ] , a member of the tumor necrosis factor  [ **TNFSF9** ]  receptor TNFR superfamily, is induced on CD4 and CD8 T cells upon engagement of the T cell receptor TCR CD3 complex with the antigen bound to MHC.  Ref: 11804328 Mol Cells, 2001
- Stimulation of 4 1BBL  [ **TNFSF9** ]  on DCs with 4 1BB  [ **TNFRSF9** ]  Fc or with 4 1BB  [ **TNFRSF9** ]  transfected Jurkat cells resulted in acquisition of capacity for the immature DCs to produce interleukin 12 IL 12.  Ref: 12590704 J Hematother Stem Cell Res, 2002
- Here, we define the molecular genetics of the human LIGHT locus, revealing its close linkage to the TNF superfamily members CD27 ligand and 4 1BB  [ **TNFRSF9** ]  ligand  [ **TNFSF9** ] , and the third complement protein C3, which positions LIGHT within the MHC paralog on chromosome 19p13.3.  Ref: 11673523 J Immunol, 2001
- ... 4 1BB  [ **TNFRSF9** ]  4 1BBL  [ **TNFSF9** ]  signaling affects T cell numbers much later in the response and is essential for the survival and or responsiveness of the memory CD8 T cell pool.  Ref: 11937529 J Immunol, 2002
- 4 1BB  [ **TNFRSF9** ]  ligand  [ **TNFSF9** ]  mediated costimulation of human T cells induces CD4 and CD8 T cell expansion, cytokine production, and the development of cytolytic effector function.  Ref: 11994439 J Immunol, 2002
- Thus, 4 1BB  [ **TNFRSF9** ]  ligand  [ **TNFSF9** ] , APRIL, CD27L, CD40L, FasL, LIGHT, TRAIL receptor 4 were upregulated, while TRAIL receptor 3 was downregulated.  Ref: 12062373 Cardiovasc Res, 2002
- In this review, we will discuss the regulatory mechanisms of TNF receptor ligand family members, such as HVEM LIGHT, 4 1BB  [ **TNFRSF9** ]  4 1BBL  [ **TNFSF9** ] , and GITR GITRL that regulate T and B cell functions and participate in the process of inflammatory diseases.  Ref: 12642898 Exp Mol Med, 2003
- We investigated whether 4 1BB  [ **TNFRSF9** ]  ligand  [ **TNFSF9** ]  4 1BBL  [ **TNFSF9** ] , a member of the tumor necrosis factor  [ **TNFSF9** ]  TNF family, is involved in the maturation process to mature myeloid DCs during in vitro DC differentiation from immature DCs derived from human umbilical cord blood CB CD34 progenitor cells.  Ref: 12590704 J Hematother Stem Cell Res, 2002
- Since the discovery of 4 1BB  [ **TNFRSF9** ]  15 years ago, the receptor and its ligand 4 1BBL  [ **TNFSF9** ]  have remained enigmatic molecules because of their highly regulated pattern of expression.  Ref: 12133793 Trends Immunol, 2002
- Expression of CD137  [ **TNFRSF9** ]  ligand 4 1BBL  [ **TNFSF9** ] , a member of the TNF family of proteins, has been reported on several types of APCs, various carcinoma cells, and can be induced on activated T cells.  Ref: 11564827 J Immunol, 2001
- CD137  [ **TNFRSF9** ]  ILA  [ **TNFRSF9** ]  4 1BB  [ **TNFRSF9** ] , a member of the tumor necrosis factor  [ **TNFSF9** ]  receptor family, and its ligand are expressed on activated T lymphocytes and on antigen presenting cells, respectively.  Ref: 11293902 Am J Clin Pathol, 2001
- Tumour rejection by gene transfer of 4 1BB  [ **TNFRSF9** ]  ligand  [ **TNFSF9** ]  into a CD80 murine squamous cell carcinoma and the requirements of co stimulatory molecules on tumour and host cells.  Ref: 11122458 Immunology, 2000
- T cell activation with systemic agonistic antibody versus local 4 1BB  [ **TNFRSF9** ]  ligand  [ **TNFSF9** ]  gene delivery combined with interleukin 12 eradicate liver metastases of breast cancer.  Ref: 12040460 Gene Ther, 2002
- Costimulation of human CD28 T cells by 4 1BB  [ **TNFRSF9** ]  ligand  [ **TNFSF9** ] .  Ref: 12645943 Eur J Immunol, 2003
- These genes included the tumor necrosis factor  [ **TNFSF9** ]  family 4 1BB  [ **TNFRSF9** ]  ligand  [ **TNFSF9** ] , ephrin B1, stra6, autotaxin, and ISLR.  Ref: 11832495 J Biol Chem, 2002
- 4 1BB  [ **TNFRSF9** ]  ligand  [ **TNFSF9** ]  induces cell division, sustains survival, and enhances effector function of CD4 and CD8 T cells with similar efficacy.  Ref: 11466348 J Immunol, 2001
